# Renal-aortic ratio as an objective measure of renal artery diameter a computed tomography angiography study

**DOI:** 10.1186/s12872-019-1163-7

**Published:** 2019-07-30

**Authors:** Marcin Majos, Michał Polguj, Ludomir Stefańczyk, Magdalena Derlatka-Kochel, Mariusz Wachowski, Agata Majos

**Affiliations:** 1 Department of Radiology and Diagnostic Imaging, Medical University of Łódź, Kopcińskiego 22, 90-153 Łódź, Poland; 2 Department of Angiology, Interfaculty Chair of Anatomy and Histology, Medical University of Lodz, Poland, Żeligowskiego 7/9, 90-752 Łódź, Poland; 3 Department of Radiological and Isotopic Diagnosis and Therapy, Medical University of Lodz, Łódź, ul. Pomorska 251, 92-213 Lodz, Poland

**Keywords:** Anatomy, Renal artery, Computed tomography, Renal-aortic ratio

## Abstract

**Background:**

Considering vital role of renal arteries in many surgical procedures, diameter of renal arteries seems to be an important measure of kidney perfusion. In this study, we analyzed a new parameter, renal-aortic ratio (R-Ar) as an objective measure of the renal artery diameter.

**Method:**

The study included CT angiographic images from 254 patients (129 women and 125 men). R-Ar was calculated by dividing the diameter of the main renal artery for each kidney by the aortic diameter.

**Results:**

R-Ar values for the whole study group ranged between 0.0863 and 0.5083; the ranges of R-Ar values for women and men patients were 0.1150–0.5083 and 0.0863–0.4449, respectively. In 412 cases (81.10%), the kidney was supplied by a single renal artery (RA variant) and in 96 (18.90%) by more than one artery (sRA variant). A significant difference was found in R-Ar values for RA and sRA variants (*p* = 0.0008). When the anatomical variant of renal perfusion was not considered on statistical analysis, a significant difference was found between the R-Ar values for women and men (*p* = 0.0259). No statistically significant difference was observed in R-Ar values for the right and left kidneys (*p* = 0.3123). Spearman’s coefficient of rank correlation between patient age and renal-aortic ratio values for the whole study group equaled − 0.36.

**Conclusion:**

The analysis of the renal-aortic ratio values demonstrated that the diameter of renal arteries depended primarily on their number, and the relative diameter of renal arteries in women was larger than in men.

## Background

A growing interest in anatomical variants of renal arteries is not surprising [1–4] considering a progress in kidney surgeries which are performed in the treatment of resistant hypertension and abdominal aortic aneurysms, or as transplantation procedures [5–7]. Modern imaging techniques can provide a detailed and highly accurate information about arterial supply of the kidneys. In this context, particularly useful seems to be computed tomography (CT) which is characterized by one of the highest spatial resolution of all radiological methods [8, 9].

Diameter of renal arteries is an important measure of kidney perfusion. Previous studies analyzed the diameters of renal arteries depending on their number and patient sex. However, the authors of those studies considered renal arteries as independent anatomical structures and did not adjust their findings for individual variance in the dimensions of the whole vascular network [10, 11].

In this study, we analyzed a new parameter, renal-aortic ratio (R-Ar) as an objective measure of renal artery diameter and its usefulness in describing vasculature. The accuracy of the R-Ar was validated in a CT angiography study.

## Method

The study included 254 patients (129 women and 125 men) subjected to CT angiography (angio-CT) at the our Department (Department of Radiology, University Clinical Hospital No. 1.) Mean age of the study participants was 66.42 years (SD = 15.07), with range between 24 and 94 years, median of 68 years and lower and upper quartile equal 58 and 78 years, respectively.

The study material included PACS-recorded images from all consecutive patients subjected to CT angiography of the abdominal aorta between January and July 2016. The inclusion criteria of the study were: presence of two normally developed kidneys without morphological evidence of pathologies with potential impact on renal artery diameter, such as atherosclerosis in a point of measurement, arterial dysplasia, dissection of arterial wall, thrombosis, etc. Patients after kidney resection (*n* = 17) or transplantation (*n* = 36), with poor quality or inadequate CT angiographic images (*n* = 20) and individuals with abdominal aortic aneurysms (*n* = 12), were excluded from the analysis.

CT angiography was performed with GE Light Speed 64 VCT scanner (GE Healthcare, Milwaukee, WI, USA; kV 120, mA 10, mAs - dynamic), with a 0.625-mm layer width and a 0.6-mm pitch, after intravenous administration of 80–100 ml of Ultravist 370 contrast agent (BAYER Schering Pharma AG, Germany) with an automatic syringe at a flow rate of 4.5 ml/s. CT angiographic images were evaluated at a GE Advantage Workstation with AW 4.0 software (GE Healthcare, Milwaukee, USA).

**Fig. 1:**
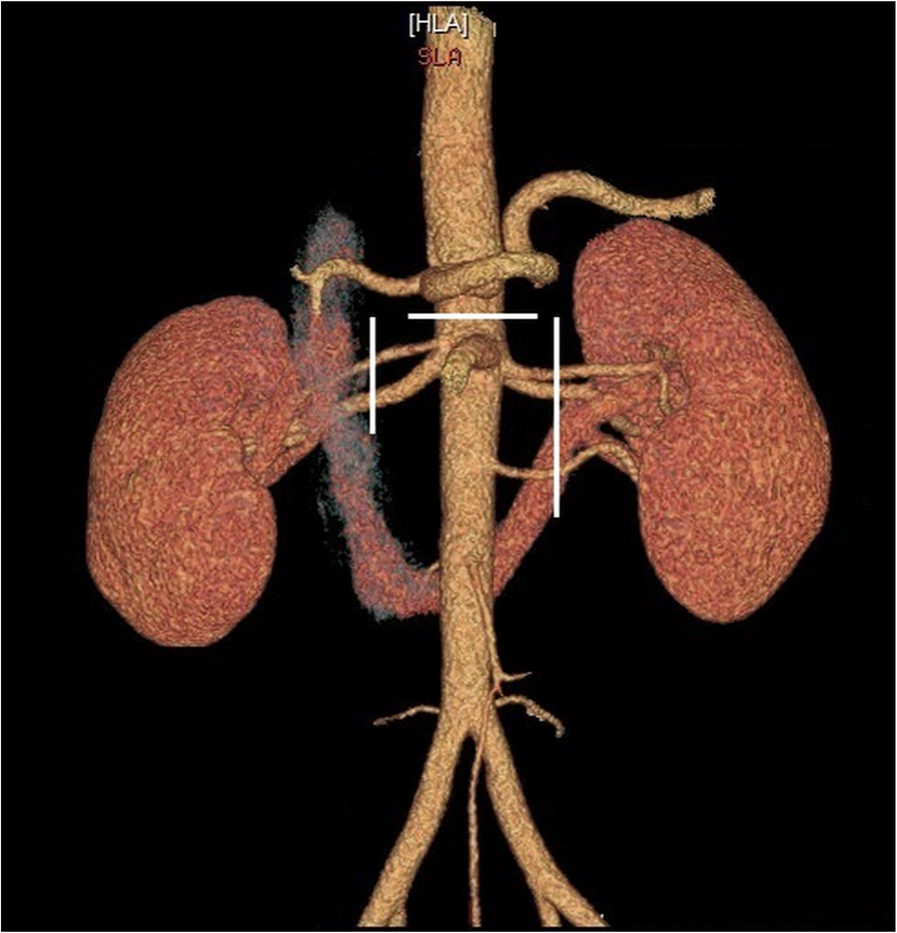
Two separated kidneys supplied by accessory renal arteries, with points where diameters were measured

The analysis included transverse and frontal images, which were used to determine the number of renal arteries on each side and to measure the diameters of either a single renal artery (RA variant) or additional renal arteries in patients whose kidneys were supplied by more than one vessel (sRA variant). The diameter of abdominal aorta was measured 5 mm above the origin of upper of the single or additional renal arteries, and the diameters of renal arteries 15 mm from their parental vessels (Fig. 1). Renal-aortic ratio (R-Ar) was calculated by dividing the diameter of the dominant renal artery by the aortic diameter. Also, renal-aortic factor (R-Af) was determined as a sum of the surface areas of the arterial lumens supplying each kidney, divided by the surface area of corresponding section of aorta with use of the following formula: $R- Af=\frac{\pi {\left(\frac{D_{R1}}{2}\right)}^2+\dots +\pi {\left(\frac{D_{Rn}}{2}\right)}^2}{\pi {\left(\frac{D_A}{2}\right)}^2}$, where (D_A_) stands for the diameter of the aorta, (D_R_) for renal arteries.

The values of R-Ar and R-Af were stratified according to the number of renal arteries and analyzed separately for kidneys supplied by a single renal artery (RA variant) and two or more arteries (sRA variant). These two groups were considered as two distinct anatomical variants of kidney perfusion. Moreover, the results were stratified according to patient sex and location of the kidney on the left or right side of the body.

Statistical characteristics of quantitative variables were presented as means, standard deviations (SD), medians, minimum and maximum values, lower and upper quartiles. Before the intergroup comparison of each quantitative variable, the normality of its distribution was verified with Shapiro-Wilk test. The significance of intergroup differences in normally distributed variables and those with distributions other than normal was verified with Student t-test and Mann-Whitney U-test, respectively. Due to lack of normal distribution in the whole study group, the direction and power of relationships between pairs of variables were analyzed based on Spearman’s coefficients of rank correlation.

Protocol of the study was approved by the Local Bioethics Committee (decision no. RNN/259/15/KE of 22 September 2016).

## Results

### Renal-aortic ratio depending on the number of renal arteries

The analysis included a total of 508 kidneys. In 412 cases (81.10%), the kidney was supplied by a single renal artery (RA variant) and in 96 (18.90%) by more than one artery (sRA variant). Statistical characteristics of R-Ar values in these two groups are presented in Table 1.

**Table 1:** Values of renal-aortic ratio for whole examined group

	N	Mean	Median	Minimum	Maximum	I kwartyl	III kwartyl	SD
Whole group	508	0.2337	0.2311	0.0863	0.5083	0.1941	0.2723	0.0591
Variant RA	412	0.2375	0.2384	0.1003	0.5083	0.1983	0.2741	0.0581
Variant sRA	96	0.2156	0.2081	0.0863	0.4449	0.1720	0.2448	0.0605

R-Ar values for RA and sRA variants differed significantly, both in the whole study group (*p* = 0.0008), and in women (*p* = 0.0203) and men patients (*p* = 0.0226).

### Renal-aortic ratio depending on patient sex

Among 258 kidneys from women, there were 220 (85.27%) and 38 (17.73%) representing RA and sRA variants, respectively. The number of kidneys from men, which represented RA and sRA variants was 192 (76.80%) and 58 (23.20%), respectively. R-Ar values for the whole study group ranged between 0.0863 and 0.5083; the ranges of R-Ar values for women and men were 0.1150–0.5083 and 0.0863–0.4449, respectively. Statistical characteristics of renal-aortic ratio values stratified according to patient sex are presented in Tables 2 and 3.

**Table 2:** Values of renal-aortic ratio for group of women

	N	Mean	Median	Minimum	Maximum	I kwartyl	III kwartyl	SD
Whole group	258	0.2392	0.2384	0.1150	0.5083	0.1987	0.2771	0.0576
Variant RA	220	0,2419	0,2438	0,0272	0,5083	0,1996	0,2773	0,0594
Variant sRA	38	0,2167	0,2088	0,1150	0,3333	0,1732	0,2358	0,0527

**Table 3:** Values of renal-aortic ratio for group of men

	N	Mean	Median	Minimum	Maximum	I kwartyl	III kwartyl	SD
Whole group	250	0.2276	0.2261	0.0863	0.4449	0.1883	0.2627	0.0601
Variant RA	192	0,231415	0,232394	0,1003	0,4032	0,1953	0,2664	0,0581
Variant sRA	58	0,214962	0,208191	0,0863	0,4449	0,1684	0,2477	0,0657

When the anatomical variant of renal perfusion was not considered on statistical analysis, a significant difference was found between R-Ar values for women and men (*p* = 0.0259). However, the sex-related difference was no longer observed when the R-Ar values for women and men were compared separately for the RA (*p* = 0.1345) and sRA variant (*p* = 0.1598).

### Renal-aortic ratio depending on body side

No statistically significant body side-related difference in R-Ar values was found, both for the whole study group (*p* = 0.3123) and after stratification of the results according to the anatomical variant of renal perfusion (*p* = 0.3902 and *p* = 0.7853 for the RA and sRA variant, respectively).

### Renal-aortic ratio for kidneys supplied by more than one renal artery (sRA variant)

Kidneys representing the sRA variant were divided into two groups: supplied by two (sRA II, *n* = 90) and three renal arteries (sRA III, *n* = 6). Mean R-Ar values for sRA II and sRA III groups were 0.2182 and 0.1736, respectively. The difference did not turn out to be statistically significant (*p* = 0.0387).

### Comparison of renal-aortic factor for kidneys supplied by a single renal artery and for kidneys supplied by additional renal vessels

Kidneys supplied by additional renal arteries were characterized with higher R-Af than these supplied only by one – 0.2243 and 0.1911, respectively. The relationship turned out to be statistically significant (*p* < 0.001). After dividing kidneys representing the RA and sRA groups according to sex, a statistically significant difference of R-Af values was found in the group of men (*p* < 0.001) only. We did not observed any significant differences in the group of women and between the sexes. (Tab. 4).

**Table 4:** Values of renal – aortic factor for whole examined group

		N	Mean	Median	Minimum	Maximum	SD
RA variant	Whole group	412	0,2243	0,1858	0,0645	0,6380	0,1151
	♀	220	0,2233	0,1851	0,0645	0,5739	0,1097
	♂	192	0,2249	0,1860	0,0802	0,6380	0,1184
sRA variant	Whole group	96	0,1911	0,1805	0,0348	0,8114	0,0909
	♀	38	0,1960	0,1874	0,0425	0,8114	0,0953
	♂	58	0,1852	0,1729	0,0348	0,5107	0,0852

### Renal-aortic ratio depending on patient age

Spearman’s coefficients of rank correlation between patient age and renal-aortic ratio values were statistically significant and equaled − 0.36 for the whole study group, and

**Fig. 2:**
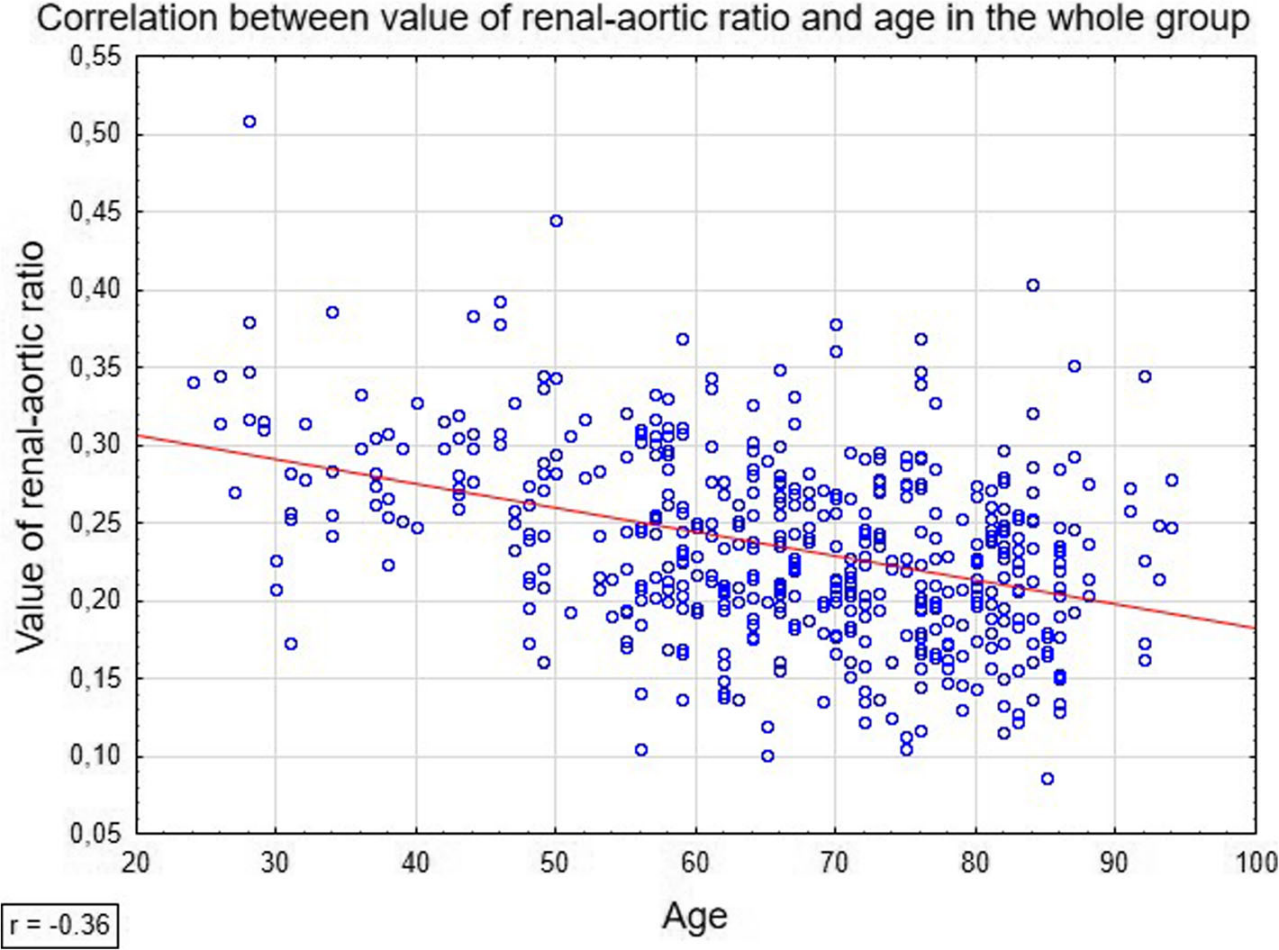
Figure demonstrates correlation between value of renal-aortic ratio and age in whole study group

− 0.43 and − 0.30 for women and men, respectively (*p* < 0.001) (Fig. 2).

## Discussion

Assessment of renal perfusion is a complex issue. Renal arteries are highly variable in terms of their number, the level they branch off the abdominal aorta, bifurcation type and diameter. These anatomical characteristics are important in the context of renal surgeries, such as partial or complete resection of the kidney [14]. However, their understanding is even more vital from the perspective of endovascular procedures. Endovascular treatment is a dynamically growing surgical discipline. It has already found application in many various indications, including management of abdominal aortic aneurysms and arterial hypertension, and with no doubt constitutes a future of modern surgery [5, 15–17]. This explains a growing demand for accurate preoperative morphological data that would facilitate the endovascular treatment. Nowadays, such data can be obtained with rapidly developing diagnostic imaging methods. Modern CT images provide very high spatial resolution, up to 0.6 mm.

However, a question arises whether the absolute values of morphometric parameters are more objective and thus, more reliable source of diagnostic information. Diameters of renal arteries are unlikely to be completely independent parameters. With no doubt, they depend on the diameters of other arteries, in particular, abdominal aorta. Thus, in this study, we proposed a new objective measure of renal artery diameter, renal-aortic ratio. This parameter was calculated based on the diameters of renal artery and abdominal aorta; we measured only the main renal artery supplying each kidney, as a pivotal vessel for its perfusion.

Our study showed that R-Ar values for kidneys supplied by a single renal artery differed significantly from those for kidneys supplied by more than two arteries. This observation is consistent with the results of our previous analysis also including absolute values of renal artery diameters [11–13]. Our findings are as well consistent with the results of previous studies conducted by other authors [1, 11, 18]. However they are not supported by results of Palmieri BJ et al. [19] and Ghabili K [20] which can be caused by measurement methods and limitations of technique of examinations.

After stratifying the study results according to patient sex, the significant difference in R-Ar values for anatomical variants with single and multiple renal arteries was found in both women and men. Also this observation is in agreement with the results of our previous study which demonstrated a significant relationship between the number and absolute diameters of renal arteries in both men and women [11].

Importantly, our present study showed that R-Ar values for kidneys from women were significantly higher than in the case of kidneys from men. In our previous study, women presented with smaller absolute diameters of renal arteries than men [10]. Due to the introduction of the relative measure, R-Ar, we demonstrated that the diameters of renal arteries in women are in fact relatively larger than in men. This observation seems to be important in the context of clinical practice.

However, the significant differences in R-Ar values for men and women were no longer observed when this parameter was analyzed separately for the RA and sRA variants. Probably the lack of statistical significance might have been associated with the disproportion in the number of men and women with the sRA variant of renal perfusion.

We showed that the values of renal-aortic ratio for right and left kidneys were essentially the same, which is consistent with our previous conclusions formulated based on absolute diameters of renal arteries.

We also analyzed in detail the values of renal-aortic ratio in the sRA group. Specifically, we compared the R-Ar values for kidneys supplied by two and three renal arteries. The difference in R-Ar values turned out to be statistically significant, which implies that the larger the number of renal arteries supplying the kidney, the smaller their diameter. However, it needs to be stressed that our series included only six kidneys that were supplied with three renal arteries, which might have been a source of analytical bias, as well that we are not determining their perfusion.

We decided also to compare renal-aortic factor to show that kidneys supplied by additional arteries are not malperfused even though they show smaller diameters. We decided to use a factor which is related to vessels’ lumen areas rather than to their diameters as the peripherical flow is slower in the vessels with laminar movement [22]. We assumed that the sum of the luminal areas of smaller, additional renal vessels needs to be equal to or slightly higher than that of a single renal artery to compensate for this phenomenon. And in fact, values of R-Af in our analysis were significantly higher in the whole sRA group than in the whole RA group as well as in the group of men, but again we did not find such a difference in the group of women.

Finally, we analyzed a relationship between the renal-aortic ratio and patient age. The relationship turned out to be statistically significant and interestingly, was markedly stronger in women than in men. Enlargement of aortic lumen with age is a physiological phenomenon [21, 23]. Our findings imply that in men, the age-related enlargement of the aorta might have been associated with a more evident increase in the diameter of renal arteries than in women. Also this observation should be considered important from a clinical perspective since men are more likely to develop aortic aneurysms than women.

## Conclusions

Introduction of renal-aortic ratio enabled us to analyze the diameters of renal arteries more objectively. Our findings support the notion that the diameter of renal arteries depends primarily on their number; this relationship was observed in both women and men. However, small size of additional renal arteries does not affect perfusion of a kidney. The relative diameters of renal arteries did not depend on the body side. Finally, we showed that the age-related relative increase in renal artery diameter was more evident in men than in women.

## Data Availability

The datasets used and/or analysed during the current study are available from the Dr. Marcin Majos (email: marcin.majos@stud.umed.lodz.pl) on reasonable request.

## Abbreviations

CTA: Computer tomography angiography
RA: Renal artery
R-Ar: Renal-aortic ratio
